# Whole-Genome Sequencing of a Haarlem Extensively Drug-Resistant *Mycobacterium tuberculosis* Clinical Isolate from Medellín, Colombia

**DOI:** 10.1128/genomeA.00566-16

**Published:** 2016-06-16

**Authors:** N. Alvarez, D. Haft, U. A. Hurtado, J. Robledo, F. Rouzaud

**Affiliations:** a Corporación para Investigaciones Biológicas–CIB, Medellín, Colombia; b J. Craig Venter Institute, Rockville, Maryland, USA; c Universidad Pontificia Bolivariana–UPB, Medellín, Colombia; d Equal Opportunity Life Sciences–EQUOLS, Rockville, Maryland, USA

## Abstract

Colombia is one of the 105 countries that has reported at least one case of extensively drug-resistant tuberculosis (XDR-TB). The *Mycobacterium tuberculosis* Haarlem genotype is ubiquitous worldwide. Here, we report the high-quality draft genome sequence of a Colombian Haarlem XDR-TB clinical isolate composed of 4,329,127 bp with 4,386 genes.

## GENOME ANNOUNCEMENT

Tuberculosis (TB) is the most lethal infectious disease worldwide, with 1.5 million annual deaths, and it is estimated that one-third of the world’s population is infected with its causative agent *Mycobacterium tuberculosis* (1). TB is a global public health concern with around 9 millions new cases per year, despite an incidence decrease by an average of 1.5% per year since 2000 (1). Colombia, with a TB incidence of 33 per 100,000 population, is one of the 105 countries that have reported at least one case of extensively drug-resistant TB (XDR-TB) to date (1). The continued spread of XDR-TB cases around the world is becoming a serious threat to public health and can be regionally illustrated by the increasingly frequent reports of XDR-TB cases in Colombia over the past few years (2–5).

The Haarlem genotype belongs to lineage 4, one of the seven major phylogeographical *M. tuberculosis* lineages that have been described around the world (6, 7). The proportion of Haarlem cases has been shown to be higher in Colombia compared to other neighboring countries, suggesting particular conditions of coevolution with the corresponding human population that favors its success (7, 8).

To better understand the molecular mechanisms involved in the extensive drug resistance of *M. tuberculosis* strains from the Haarlem genotype, we sequenced the whole genome of a clinical isolate from Medellín, Colombia.

In this study, 24-locus mycobacterial interspersed repetitive-unit–variable-number tandem-repeat analysis was used to confirm the assignment of isolate TBR-102 to the *M. tuberculosis* Haarlem sublineage (9, 10). Phenotypic susceptibility tests to first- and second-line drugs were performed using the BACTEC MGIT 960 method. It was determined that TBR-102 is resistant to isoniazid, rifampin, ofloxacin, and amikacin and is therefore classified as an XDR-TB. Genomic DNA was purified from the isolate by the CTAB method (11). Samples were treated with RNase prior to paired-end library construction with an average insert target size of 800 bp. Whole-genome sequencing was performed at the J. Craig Venter Institute in Rockville, Maryland, USA, on an Illumina MiSeq platform with 250-bp reads and to about 70× coverage. The draft whole-genome sequences are composed of 4,329,127 bp with a 65% GC content. A *de novo* assembly was carried out using Celera Assembler (12), and structural and functional annotation was completed using multiple-ranked sources of evidence, including the TIGRFAMs (13) and Pfam (14) protein family databases. The assembled sequence reveals 4,386 protein-coding genes, along with 45 tRNAs. Mutations associated with resistance to rifampin (D435V in *rpoB*) and isoniazid (S315T in *katG*) were encountered. Also, TBR-102 displayed the *rrs* gene A1401C substitution associated with resistance to aminoglycosides, as well as the *gyrA* D94G mutation associated with resistance to fluoroquinolones. No mutations were detected in *gyrB*.

This report of a Haarlem XDR-TB Colombian isolate genome will provide material for comparative genomic analysis with XDR-TB genomes circulating in Colombia, Latin America, and other regions of the world (15–19).

### Nucleotide sequence accession numbers.

This whole-genome shotgun project has been deposited at DDBJ/ENA/GenBank under the accession number JRJS00000000. The version described in this paper is the first version, JRJS01000000.
